# Glucagon-like Peptide-1 Receptor Agonists and Suicidal Ideation: Analysis of Real-Word Data Collected in the European Pharmacovigilance Database

**DOI:** 10.3390/ph17020147

**Published:** 2024-01-23

**Authors:** Rosanna Ruggiero, Annamaria Mascolo, Angela Spezzaferri, Claudia Carpentieri, Daniele Torella, Liberata Sportiello, Francesco Rossi, Giuseppe Paolisso, Annalisa Capuano

**Affiliations:** 1 Campania Regional Centre for Pharmacovigilance and Pharmacoepidemiology, 80138 Napoli, Italy; rosanna.ruggiero@unicampania.it (R.R.);; 2 Department of Experimental Medicine—Section of Pharmacology “L. Donatelli”, University of Campania “Luigi Vanvitelli”, 80138 Napoli, Italy; 3 Department of Experimental and Clinical Medicine, Magna Græcia University, 88100 Catanzaro, Italy; dtorella@unicz.it; 4 Department of Advanced Medical and Surgical Sciences, University of Campania “Luigi Vanvitelli”, 80138 Napoli, Italy; 5 UniCamillus International Medical University, 00131 Rome, Italy

**Keywords:** retrospective study, pharmacovigilance, safety, glucagon-like peptide-1 receptor agonists, suicidal event, disproportional reporting

## Abstract

Background: A potential risk of suicide associated with liraglutide or semaglutide treatments has recently emerged. Therefore, we decided to investigate the reporting probability of suicidal events among glucagon-like peptide-1 receptor agonists (GLP-1 RAs). Methods: A retrospective pharmacovigilance study of the European Pharmacovigilance database was conducted for the period from 1 January 2018 to 10 July 2023. Disproportionality analyses (reporting odds ratio, ROR) were performed to assess the reporting probability of suicidal events among GLP-1 RAs. Results: A total of 230 reports of suicidal events were identified. The most reported GLP-1 RA was liraglutide (38.3%), followed by semaglutide (36.5%) and dulaglutide (16.1%). The most reported events were suicidal ideation (65.3%) and suicide attempt (19.5%). Disproportionality analysis found a higher reporting probability of suicidal events for semaglutide than dulaglutide (ROR, 2.05; 95%CI, 1.40–3.01) and exenatide (ROR, 1.81; 95%CI, 1.08–3.05). In the same way, liraglutide was associated with a higher reporting probability of suicidal events than dulaglutide (ROR, 3.98; 95%CI, 2.73–5.82) and exenatide (ROR, 3.52; 95%CI, 2.10–5.92). On the contrary, a lower reporting probability was found for semaglutide than liraglutide (ROR, 0.51; 95%CI, 0.38–0.69). Conclusions: Suicidal events were mostly reported with semaglutide and liraglutide, which were also associated with significantly higher reporting probabilities compared to other GLP1 RAs. Although this study provides the reporting frequencies of suicide-related events with GLP-1 RAs, establishing causality requires further investigation, which will probably be addressed by the Pharmacovigilance Risk Assessment Committee of the European Medicine Agency in the future.

## 1. Introduction

On 11 July 2023, the European Medicine Agency (EMA) started an ongoing safety revision of glucagon-like peptide-1 receptor agonists (GLP-1 RAs), which is being carried out by the EMA Pharmacovigilance Risk Assessment Committee (PRAC) [1]. In particular, the data review focuses on the risk of suicidal thoughts and thoughts of self-harm in patients treated with these drugs.

GLP-1 RAs are incretin-mimetic agents, being mainly recommended for type 2 diabetes mellitus (T2DM) [2]. By stimulating GLP-1 receptors, this class of drugs can lower blood glucose, increase insulin secretion induced by hyperglycemia, and suppress glucagon release in hyper- or euglycemia. To date, in Europe, six GLP-1 RAs have been authorized. Exenatide was the first one, being authorized in 2006 for T2DM. Subsequently, the European Agency approved liraglutide (in 2009), lixisenatide (in 2013), combination liraglutide/insulin degludec, and dulaglutide (both in 2014), and most recently, semaglutide (in 2018). Another GLP-1 RA, albiglutide, was withdrawn from the European market upon request of the marketing authorization holder in 2018 for commercial reasons. All GLP-1 RAs are administered subcutaneously. The new oral semaglutide formulation, introduced in 2020, represents a pharmaceutical innovation aimed at increasing compliance in diabetic patients. Moreover, GLP-1 RAs have demonstrated cardiovascular benefits in high-risk patients, such as those with coronary syndromes, heart failure, or chronic kidney diseases, representing pharmacological progress in reducing the burden not only of diabetes mellitus but also of its complications [3]. The most common adverse events of GLP-1 RAs are gastrointestinal symptoms (nausea, vomiting, diarrhoea), which occur at start of the treatment and during dose escalation. Therefore, to reduce the onset of gastrointestinal events, gradual up-titration should be performed [3].

Beyond hypoglycaemic, endocrine, and cardiovascular effects, several decades of research have also shown the extended actions of GLP-1 RAs, which include decelerated gastric emptying and a reduction in food intake and body weight [4]. These are due to the expression of GLP-1 receptors in the central nervous system, including the area of the brain that regulates appetite [5]. For this reason, GLP-1 RAs increase feelings of fullness and decrease feelings of hunger. These additional actions have promoted their use for body weight control. Indeed, two GLP-1 RAs (liraglutide and semaglutide) were authorized in 2015 and 2022, respectively, for obesity or overweight treatment. In particular, Saxenda^®^ (liraglutide) and Wegovy^®^ (semaglutide) were approved as adjuncts to a reduced-calorie diet and increased physical activity for weight management in adults with a body mass index (BMI) of ≥30 kg/m^2^ (obesity) or ≥27 kg/m^2^ to <30 kg/m^2^ (overweight) in the presence of at least one weight-related comorbidity (e.g., prediabetes, type 2 diabetes mellitus, hypertension, dyslipidaemia, obstructive sleep apnoea, or cardiovascular disease) and as an adjunct to healthy nutrition and increased physical activity for weight management in obese adolescents (≥12 years) with a BMI corresponding to 30 kg/m^2^ for adults or with body weight above 60 kg. 

Recently, the increased demand for semaglutide and the possible misuse of these antidiabetics as a weight loss treatment in non-obese people have been described in the literature and by the media [6,7,8]. Almost simultaneously, reports of suicidal thoughts in people using liraglutide and semaglutide were highlighted [1]. For this reason, the PRAC started a safety review initially involving semaglutide and liraglutide and subsequently expanded it to the entire class of GLP-1 RAs [1]. The evidence evaluating this risk is meagre. Few recent studies have questioned this association [9,10,11], and no study has compared this risk between different GLP-1 RAs. Clinical trials have several limitations related to their duration and patient inclusion criteria that can impede investigations on psychiatric adverse events. Consequently, psychiatric safety issues may emerge from clinical practice through the analysis of real-world safety data [12]. Considering the clinical relevance of this potential suicidal risk associated with GLP-1 RAs and the availability of pharmacovigilance databases as a source of real-world information, the present study aimed to evaluate the characteristics of suicidal events reported with GLP-1 RAs through the analysis of the pharmacovigilance database EudraVigilance (EV).

## 2. Results

### 2.1. Descriptive Characteristics of Individual Case Safety Reports

During the period from 1 January 2018 to 10 July 2023, a total of 41,236 Individual Case Safety Reports (ICSRs) related to GLP-1 RAs were retrieved from EV, of which 230 (0.6%) reported at least one suicidal event. Descriptive characteristics for each GLP1 RA are listed in Table 1. The most reported GLP1 RA was liraglutide (*n* = 88; 38.3%), followed by semaglutide (*n* = 84; 36.5%), dulaglutide (*n* = 37; 16.1%), exenatide (*n* = 16; 6.9%), and liraglutide/insulin degludec (*n* = 5; 2.2%). For semaglutide, 9 ICSRs (10.7%) were related to the oral formulation (Rybelsus^®^) and 11 (13.1%) to the formulation authorized for weight management (Wegovy^®^). For liragluride, instead, 60 ICSRs (68.2%) were related to the formulation authorized for weight management (Saxenda^®^). No ICSR of suicide with lixisenatide was reported in EV. Considering that more than one suspected drug can be reported in an ICSR, we found that ICSRs of suicide reported a single GLP-1 RA as a suspected drug. Among ICSRs, the most reported age group was 18–64 years (*n* = 134; 58.3%), and the most reported sex was female (*n* = 133; 57.8%). The primary reporter was the healthcare professional (*n* = 151; 65.7%), and most ICSRs came from the non-European Economic Area (*n* = 191; 83.0%). The therapeutic indication was not reported for most ICSRs (*n* = 112; 48.7%), but when available, it was obesity/weight control (*n* = 48; 20.9%) and diabetes/blood glucose control (*n* = 67; 29.1%). Specifically, the most reported indications were diabetes mellitus (*n* = 63; 27.4%) and weight control (*n* = 32; 13.9%). ICSRs mainly reported diabetes/blood glucose control for dulaglutide (*n* = 24) and semaglutide (*n* = 20), while obesity/weight control for liraglutide (*n* = 30) and semaglutide (*n* = 14), with only one ICSR reported with exenatide. The GLP1 RA was the only suspected drug reported in most ICSRs (*n* = 166; 72.2%), and no concomitant medication was reported (*n* = 152; 66.1%).

A total of 124 other suspected drugs were identified in ICSRs with more suspected drugs (*n* = 64; 27.8%), (Supplementary Table S1). Specifically, most suspected drugs belonged to ATC A10A (insulins and analogues (*n* = 27; 21.8%)), followed by N06A (antidepressants (*n* = 14; 11.3%)), A10B (blood-glucose-lowering drugs (*n* = 12; 9.7%)), and N03A (antiepileptics (*n* = 11; 8.9%)). All other ATCs of suspected drugs and a list of active ingredients are shown in Supplementary Tables S1 and S2, respectively. 

A total of 78 (33.9%) ICSRs reported one or more concomitant drugs for a total of 363 concomitants. The most reported concomitants belonged to the ATC A10B (blood-glucose-lowering drugs (*n* = 49; 13.5%)), N06A (antidepressants (*n* = 35; 9.6%), C10A (lipid-modifying agents, plain (*n* = 25; 6.9%)), and A02B (drugs for peptic ulcer and gastro-oesophageal reflux disease (*n* = 16; 4.4%)). All other ATCs of concomitant drugs and a list of active ingredients are shown in Supplementary Tables S3 and S4, respectively.

### 2.2. Descriptive Characteristics of Suicidal Events

A total of 236 suicidal events out of 230 ICSRs were identified (Table 2). The most reported events were suicidal ideation (*n* = 154; 65.3%) and suicide attempt (*n* = 46; 19.5%). Suicidal ideation was primarily reported with liraglutide (60 out of 90 suicidal events; 66.7%) and semaglutide (67 out of 86; 77.9%), while suicide attempts were primally observed with dulaglutide (15 out of 38; 39.5%) and liraglutide (16 out of 90; 17.8%). Both events were mainly reported in females (suicidal ideation: 99 out of 137 events in females, 72.3%; suicide attempt: 22 out of 137; 16.1%; Table 2). All suicidal events (except for one unknown) were serious, with the most reported seriousness criterion being “other medically important condition” (*n* = 161; 68.2%). The outcome was unavailable for 106 ICSRs (44.9%) and resolved in 74 ICSRs (31.4%). The outcome was fatal in 14 ICSRs (5.9%). All seriousness and outcome criteria for each GLP1 RA are listed in Table 3.

### 2.3. Disproportionality Analysis

**Figure 1:**
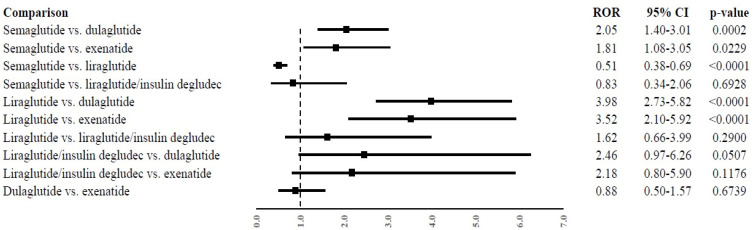
Reporting probabilities of suicidal events among GLP-1 receptor agonist. Data are expressed as reporting odds ratios (RORs) and their 95% confidence intervals (95%CI).

From the disproportionality analysis, statistically significant differences emerged for semaglutide or liraglutide compared to other GLP1 RAs. In particular, the probability of reporting suicidal events was found to be higher for semaglutide than dulaglutide (ROR, 2.05; 95%CI, 1.40–3.01) or exenatide (ROR, 1.81; 95%CI, 1.08–3.05) and higher for liraglutide than dulaglutide (ROR, 3.98; 95%CI, 2.73–5.82) or exenatide (ROR, 3.52; 95%CI, 2.10–5.92). On the contrary, the reporting probability was found to be lower for semaglutide than liraglutide (ROR, 0.51; 95%CI, 0.38–0.69). All other comparisons did not show statistically significant differences (Figure 1).

## 3. Discussion

The present study is the first pharmacovigilance study to evaluate the reporting of suicidal events as suspected adverse drug reactions associated with the GLP1 RAs, by using the European pharmacovigilance database. GLP1 RA-induced suicidal events were mentioned in the European risk management plans for such medicines. They were brought to the attention of the Icelandic Medicines Agency after the reporting of cases of suicidal thoughts and self-harm in people using liraglutide and semaglutide [1]. This study provides reporting frequencies of suicide-related events with GLP-1 RAs. However, the causation requires further investigation, and a conclusion will be provided by the PRAC in the future. In this study, we found a 2-fold increase in the reporting of suicidal events with semaglutide compared with exenatide and dulaglutide. This estimate was even higher in the comparison between liraglutide and exenatide or dulaglutide, with 4- and 3.5-fold increases in the reporting of suicidal events, respectively. Moreover, when the comparison was between semaglutide and liraglutide, semaglutide showed a lower reporting of suicidal events. The more recent marketing authorization of semaglutide may explain this finding and the lower reporting of suicidal events. Both medicines also have the central effect of reducing appetite and are used not only for the treatment of diabetes mellitus but also for the treatment of obesity and overweight [13,14]. Generally, all centrally acting anti-obesity drugs draw attention for their neuropsychiatric safety [15,16]. A pooled post hoc analysis of neuropsychiatric safety data from the liraglutide weight management clinical trials program on liraglutide 3.0 mg found that the incidences of depression, anxiety, and insomnia were low (≤4%) in both the liraglutide and placebo groups, but with a slight increase in insomnia and suicidal ideation for liraglutide [17]. On the contrary, clinical trials with lower doses of liraglutide (up to 1.8 mg) for T2DM did not find any safety signal for neuropsychiatric events [18,19]. Therefore, it seems that the suicidal risk of GLP1 RAs could be related to the use of higher doses for weight management. Indeed, based on this consideration, the US prescribing information already states that patients should be monitored during treatment with liraglutide 3.0 mg for depression or suicidal thoughts and should discontinue the drug if these symptoms appear [20]. The same warning is also reported in the US prescribing information of semaglutide for obesity (Wegovy^®^), which is also used at higher dosages for weight control than T2DM [21]. However, these warnings are not reported in any European summary of the product characteristics (SmPCs) for any GLP-1 RA.

A possible mechanism explaining the risk of suicidal events with GLP-1 RAs is related to their action in the hypothalamus [5] since the hyperactivity of the hypothalamic–pituitary–adrenal axis has been associated with suicidal behaviours [22]. However, the evidence for the role of GLP1 in the nervous system is conflicting. There is also evidence showing the ability of GLP1 to attenuate neuroinflammation, protect neurons and glia from oxidative stress, and improve neurotransmitter balance [23]. Moreover, the potential interaction between GLP-1 and serotonin pathways has been the objective of recent studies for energy balance regulation [24,25]. We cannot exclude the possibility that this interaction may also play a role in the pathogenesis of depression and suicidal ideation. Therefore, any causal conclusion is even more complicated. Indeed, many risk factors may contribute to or precipitate the onset of suicidal behaviours in patients treated with GLP1 RAs, including psychosocial and biological factors.

Considering that suicide is a public health problem, the identification of risk factors is fundamental, representing the fourth leading cause among the 15–29-years-old group. According to the latest World Health Organization (WHO) data, more than 700,000 people die each year due to suicide [22]. Suicides require timely, evidence-based, and adequate interventions based on the identified risk factors [26]. 

Multiple biological factors can also contribute to the pathophysiology of adverse events [27,28], including suicidal events [29]. Among these, sex can contribute to suicidal behaviours. In the literature, suicide attempts are more frequent in females, while completed suicides are three times more common in males [22]. In line with this, in our analysis, females more frequently described suicidal ideation and suicide attempts in ICSRs, while the rate of completed suicide was three times higher in males than females. Moreover, underlying or concomitant diseases and concomitant drug treatments are possible confounding factors for suicide. Several diseases and pharmacological treatments can increase suicide risk. First of all, diabetic and obese patients represent a fragile population, being more exposed to the risk of suicide than the general population because of their underlying pathologies [30]. Both diabetes [31,32] and obesity [33,34] have been identified as suicidal risk factors. Among obese patients, the association between obesity and suicide seems to be influenced by sex, being greater for women than men [35]. Moreover, suicidal ideation could also be a consequence of the therapeutic inefficacy of GLP1 RAs used for weight control. A cross-sectional study suggested an association between weight control failure and suicidal ideation in overweight and obese adults, especially in women [36]. However, in our dataset, no ICSRs reported therapeutic ineffectiveness. Regarding diabetes, both types of diabetes were associated with an increased occurrence of psychiatric disorders, including suicidal ideation. Psychiatric disorders, in particular depression, are well-known suicide risk factors [22]. In our dataset, antidepressants were the most reported suspected as well as concomitant drugs. Moreover, most suspected drugs were also antiepileptics. Even if a recent meta-analysis seems to question it [37], the link between suicide and these medications is widely described in the literature [38,39,40] and reported in all SmPCs of these drugs. Finally, other suspected and concomitant medicines reported in our ICSRs may have influenced the development of symptoms of suicidal behaviour. Statins, for example, have been associated with higher reporting of psychiatric adverse events in a previous pharmacovigilance study [41]. Proton pump inhibitors have also been investigated for their psychiatric effects, including suicidal ideation and depression [42]. Renin–angiotensin–aldosterone system (RAAS) blockers have also been found to be related to the risk of suicide due to the central role played by RAAS in mood disorders [43]. Considering the many contributing risk factors for suicide, understanding its causal relationship with medicine may be very hard, thus requiring further research on this topic. 

The main strength of our study is the use of a broad data source, the EV database, which allows the evaluation of safety cases all over Europe. Moreover, a pharmacovigilance database has a low cost and is helpful for characterizing drug safety profiles in the real world. However, this study also has several limitations. First of all, some cases of suicide with GLP1 RAs may not have been reported to the national drug authorities and thus not submitted to EV (the underreporting phenomenon). Underreporting is a notable limitation of pharmacovigilance systems, as only 6–10% of all adverse events are reported to regulatory authorities [44,45]. This underreporting can prevent the quantification of the incidence of adverse events and the risk estimates [45], and it can delay the identification of safety signals, with repercussions for public health [45,46,47]. Another limitation is the quality of information reported. Indeed, ICSRs may be incomplete and lacking useful clinical information, such as clinical history and concomitant comorbidities and medications. This lack of information can impede the evaluation of confounding factors. From our data source, we cannot evaluate data on lifestyle factors, psychiatric comorbidities, or other medical conditions that could be associated with the risk of suicide. We also cannot retrieve information on the exact dates of administration and event onset. Both underreporting of ICSRs and possibly missing information can introduce information biases in the analysis. Moreover, the exact number of patients exposed to GLP1 RAs is unavailable in EV (real users), where we can only use the total number of events of each drug as a denominator for disproportionality analyses. Finally, our analyses were restricted to comparisons within the drug class of GLP1 RAs. Considering these limitations, our study only aimed to analyse ICSRs related to suicide with GLP1 RAs and to show the reporting probability of these adverse events but refrained from asserting any direct causal association between GLP-1 RAs and the risk of suicidal events.

## 4. Materials and Methods

### 4.1. Study Design

This was a retrospective European pharmacovigilance study aiming to compare the reporting probability of suicidal events between GLP1 RAs.

### 4.2. Data Source

The EV is the European pharmacovigilance database, managed by the EMA, used for the management, collection, and analyses of all adverse events that, according to the reporters’ opinion, may be potentially related to the administration of a drug/s or vaccine/s. Therefore, it collects all safety reports sent by healthcare professionals or citizens to medicines regulatory authorities that describe any adverse drug reaction/s (ADRs) or adverse events following immunization (AEFIs) [28]. The analysis of pharmacovigilance databases is included in the continuous monitoring of medicines, allowing the extrapolation of safety information and signals from the real-life context [48,49]. According to a transparency policy, the data collected in the EV are publicly available on the EMA website (www.adrreports.eu, accessed on 13 July 2023).

### 4.3. Data Retrieval

On 13 July 2023, we retrieved from the EMA website (www.adrreports.eu) all ICSRs reporting at least one GLP-1 RA as a suspected drug and collected in EV from 1 January 2018 (EMA authorization year of the most recent GLP-1 RA semaglutide) to 10 July 2023. To identify all GLP1 RAs, we used the Anatomical Therapeutic Chemical (ATC) Classification A10BJ, which includes semaglutide, liraglutide, exenatide, lixisenatide, dulaglutide, albiglutide, and beinaglutide. From our research, we excluded the following molecules: albiglutide, which was actually withdrawn from the European market due to commercial reasons [50], and beinaglutide, which his authorized only in the United States [51] and China [52]. We also included the combination product liraglutide/insulin degludec.

### 4.4. Data Management

ICSRs were downloaded from the EMA website by interrogating this tool with a level of access dedicated to the stakeholder group II (SGII: healthcare professionals and the public) based on the revised Eudravigilance access policy. ICSRs were retrieved as Microsoft Excel files for each GLP1 RA, in which a row is an ICSR. Then, all extracted Excel files were merged. and duplicates were removed.

### 4.5. Descriptive Analysis

We focused our analysis on ICSRs reporting a GLP-1 RA and a suicidal event as an ADR. According to the *Medical Dictionary for Regulatory Activities* (MedDRA) version 26.1, suicidal events were identified based on the Preferred Terms: completed suicide, depression suicidal, suicidal behaviour, suicidal ideation, or suicide attempt. We conducted a descriptive analysis of these ICSRs, describing their demographic characteristics. In particular, we described the age group and sex of patients, the type of reporter (as healthcare professional or non-healthcare professional), the country source (European Economic Area or non-European Economic Area). These data are collected in an ICSR through the spontaneous reporting system, whereby a healthcare professional or citizen can report a suspected adverse drug reaction to a National Competent Authority. Moreover, we described the number of concomitant or suspected drugs and the therapeutic indication of the GLP-1 RA reported as a suspected drug. Subsequently, we described the reported suicidal events in terms of outcome and seriousness criteria for each GLP1 RA and for the sex of patients. The outcome of suicidal events was classified as “recovered/resolved”, “recovering/resolving”, “recovered/resolved with sequelae”, “not recovered/not resolved”, “fatal”, and “unknown”. According to the current pharmacovigilance regulations, seriousness criteria included “results in death”, “caused/prolonged hospitalization”, “disabling”, “life-threatening”, “congenital abnormalities/birth deficits”, or “other medically important condition”. Finally, we described other suspected drugs reported in ICSRs of suicidal events by specifying the active ingredients and classifying them by the ATC Classification.

### 4.6. Disproportionality Analysis

To identify a hypothetical different reporting probability of suicidal events between GLP1 RAs, we performed a disproportionality analysis by computing the reporting odds ratio (ROR) and its 95% confidence interval (95%CI) of suicidal events among GLP-1 RAs. The following comparisons were performed: semaglutide vs. dulaglutide, or exenatide, or liraglutide, or liraglutide/insulin degludec; liraglutide vs. dulaglutide, or exenatide, or liraglutide/insulin degludec; liraglutide/insulin degludec vs. dulaglutide or exenatide; dulaglutide vs. exenatide. The ROR was calculated as (a/c)/(b/d), where “a” is the number of suicidal events reported with a GLP1 RA, “c” is the number of suicidal events reported with the comparator (a different GLP1 RA), “b” is the number of other events reported with a GLP1 RA, and “d” is the number of other events reported with the comparator. A signal was identified when suicidal events were reported at least 5 times, and the lower bound of the 95%CI was higher than 1. For statistical significance, a *p*-value < 0.05 was considered.

### 4.7. Ethical Statement

Safety data deriving from pharmacovigilance databases are anonymous and in compliance with the ethical standard. Therefore, no further ethical measure was required.

## 5. Conclusions

Our study found 230 ICSRs describing suicidal events with GLP1 RAs. The most reported drugs were semaglutide and liraglutide, for which the disproportionality analyses found higher reporting probabilities than other GLP1 RAs. Among semaglutide and liraglutide ICSRs, 13.1% and 68.2% were related to the formulation authorized for weight management, respectively. The most reported events were suicidal ideation and suicide attempts in females and completed suicide in men. However, considering all the possible contributing factors for suicide, further investigations that take into account these aspects are needed, because the clinical trials were not powered enough to evaluate specifically neuropsychiatric events. This research underscores the need for strictly adhering to evidence-based practices when prescribing GLP1 RAs. Moreover, it highlights the importance of strengthening pharmacovigilance activities to facilitate the availability of more data concerning GLP-1 RAs in the future.

## Figures and Tables

**Table 1:** Demographic characteristics (age, sex, type of reporter, country source, and number of concomitant or suspected drugs) of Individual Case Safety Reports (ICSRs) reporting suicidal events associated with dulaglutide, exenatide, liraglutide, liraglutide/insulin degludec, or semaglutide as suspected drugs.

	Dulaglutide (*n* = 37)	Exenatide (*n* = 16)	Liraglutide (*n* = 88)	Liraglutide/Insulin Degludec (*n* = 5)	Semaglutide (*n* = 84)	Overall (*n* = 230)
Age group						
12–17 years	0 (0%)	1 (6.3%)	5 (5.7%)	0 (0%)	0 (0%)	6 (2.6%)
18–64 years	19 (51.4%)	5 (31.3%)	54 (61.4%)	4 (80.0%)	52 (61.9%)	134 (58.3%)
65–85 years	10 (27.0%)	2 (12.5%)	9 (10.2%)	1 (20.0%)	4 (4.8%)	26 (11.3%)
Not specified	8 (21.6%)	8 (50.0%)	20 (22.7%)	0 (0%)	28 (33.3%)	64 (27.8%)
Sex						
Female	18 (48.6%)	7 (43.8%)	60 (68.2%)	0 (0%)	48 (57.1%)	133 (57.8%)
Male	17 (45.9%)	9 (56.3%)	28 (31.8%)	5 (100%)	29 (34.5%)	88 (38.3%)
Not specified	2 (5.4%)	0 (0%)	0 (0%)	0 (0%)	7 (8.3%)	9 (3.9%)
Reporter						
Healthcare professional	26 (70.3%)	5 (31.3%)	64 (72.7%)	5 (100%)	51 (60.7%)	151 (65.7%)
Non-healthcare professional	11 (29.7%)	11 (68.8%)	24 (27.3%)	0 (0%)	33 (39.3%)	79 (34.3%)
Country						
European Economic Area	6 (16.2%)	0 (0%)	14 (15.9%)	3 (60.0%)	16 (19.0%)	39 (17.0%)
Non-European Economic Area	31 (83.8%)	16 (100%)	74 (84.1%)	2 (40.0%)	68 (81.0%)	191 (83.0%)
Concomitant drugs						
0	22 (59.5%)	2 (12.5%)	62 (70.5%)	2 (40.0%)	64 (76.2%)	152 (66.1%)
1	7 (18.9%)	0 (0%)	5 (5.7%)	1 (20.0%)	5 (6.0%)	18 (7.8%)
2	3 (8.1%)	0 (0%)	10 (11.4%)	0 (0%)	5 (6.0%)	18 (7.8%)
3	3 (8.1%)	3 (18.8%)	5 (5.7%)	0 (0%)	1 (1.2%)	12 (5.2%)
4	0 (0%)	6 (37.5%)	0 (0%)	0 (0%)	2 (2.4%)	8 (3.5%)
5	2 (5.4%)	5 (31.3%)	6 (6.8%)	2 (40.0%)	7 (8.3%)	22 (9.6%)
Suspected drugs						
1	21 (56.8%)	7 (43.8%)	62 (70.5%)	1 (20.0%)	75 (89.3%)	166 (72.2%)
2	11 (29.7%)	5 (31.3%)	18 (20.5%)	4 (80.0%)	7 (8.3%)	45 (19.6%)
3	3 (8.1%)	0 (0%)	2 (2.3%)	0 (0%)	2 (2.4%)	7 (3.0%)
4	2 (5.4%)	0 (0%)	0 (0%)	0 (0%)	0 (0%)	2 (0.9%)
5	0 (0%)	4 (25.0%)	6 (6.8%)	0 (0%)	0 (0%)	10 (4.3%)
GLP1 RA ^1^ therapeutic indication						
Diabetes mellitus	24 (64.9%)	7 (43.8%)	9 (10.2%)	3 (60.0%)	20 (23.8%)	63 (27.4%)
Unknown	13 (35.1%)	5 (31.3%)	47 (53.4%)	1 (20.0%)	46 (54.8%)	112 (48.7%)
Blood glucose control	0 (0%)	3 (18.8%)	1 (1.1%)	0 (0%)	0 (0%)	4 (1.7%)
Weight control	0 (0%)	1 (6.3%)	19 (21.6%)	0 (0%)	12 (14.3%)	32 (13.9%)
Obesity	0 (0%)	0 (0%)	10 (11.4%)	0 (0%)	2 (2.4%)	12 (5.2%)
Polycystic ovaries, weight control	0 (0%)	0 (0%)	1 (1.1%)	0 (0%)	0 (0%)	1 (0.4%)
Suicide attempt	0 (0%)	0 (0%)	1 (1.1%)	1 (20.0%)	0 (0%)	2 (0.9%)
Diabetes mellitus, overweight/Obesity	0 (0%)	0 (0%)	0 (0%)	0 (0%)	3 (3.6%)	3 (1.3%)
Polycystic ovaries	0 (0%)	0 (0%)	0 (0%)	0 (0%)	1 (1.2%)	1 (0.4%)

^1^ Glucagon-like peptide-1 receptors agonists (GLP1 RAs).

**Table 2:** Suicidal events distributed for dulaglutide, exenatide, liraglutide, liraglutide/insulin degludec, and semaglutide and sex.

	Dulaglutide (*n* = 38)	Exenatide (*n* = 17)	Liraglutide (*n* = 90)	Liraglutide/Insulin Degludec (*n* = 5)	Semaglutide (*n* = 86)	Overall (*n* = 236)	Female (*n* = 137)	Male (*n* = 90)	Not Specified (*n* = 9)
Events									
Completed suicide	2 (5.3%)	1 (5.9%)	5 (5.6%)	0 (0%)	3 (3.5%)	11 (4.7%)	2 (1.5%)	9 (10.0%)	0 (0%)
Depression suicidal	2 (5.3%)	1 (5.9%)	5 (5.6%)	0 (0%)	9 (10.5%)	17 (7.2%)	11 (8.0%)	6 (6.7%)	0 (0%)
Suicidal behaviour	2 (5.3%)	2 (11.8%)	1 (1.1%)	0 (0%)	0 (0%)	5 (2.1%)	2 (1.5%)	3 (3.3%)	0 (0%)
Suicidal ideation	17 (44.7%)	10 (58.8%)	60 (66.7%)	0 (0%)	67 (77.9%)	154 (65.3%)	99 (72.3%)	47 (52.2%)	8 (88.9%)
Suicide attempt	15 (39.5%)	3 (17.6%)	16 (17.8%)	5 (100%)	7 (8.1%)	46 (19.5%)	22 (16.1%)	23 (25.6%)	1 (11.1%)
Suspected suicide	0 (0%)	0 (0%)	3 (3.3%)	0 (0%)	0 (0%)	3 (1.3%)	1 (0.7%)	2 (2.2%)	0 (0%)

**Table 3:** Seriousness and outcome criteria of suicidal events reported with dulaglutide, exenatide, liraglutide, liraglutide/insulin degludec, and semaglutide.

	Dulaglutide (*n* = 38)	Exenatide (*n* = 17)	Liraglutide (*n* = 90)	Liraglutide/Insulin Degludec (*n* = 5)	Semaglutide (*n* = 86)	Overall (*n* = 236)
Seriousness Criteria						
Caused/prolonged hospitalization	10 (26.3%)	5 (29.4%)	10 (11.1%)	4 (80.0%)	8 (9.3%)	37 (15.7%)
Life-threatening	3 (7.9%)	4 (23.5%)	6 (6.7%)	1 (20.0%)	6 (7.0%)	20 (8.5%)
Other medically important condition	23 (60.5%)	7 (41.2%)	63 (70.0%)	0 (0%)	68 (79.1%)	161 (68.2%)
Results in death	2 (5.3%)	1 (5.9%)	8 (8.9%)	0 (0%)	3 (3.5%)	14 (5.9%)
Not reported	0 (0%)	0 (0%)	1 (1.1%)	0 (0%)	0 (0%)	1 (0.4%)
Disabling	0 (0%)	0 (0%)	2 (2.2%)	0 (0%)	1 (1.2%)	3 (1.3%)
Outcome						
Fatal	2 (5.3%)	1 (5.9%)	8 (8.9%)	0 (0%)	3 (3.5%)	14 (5.9%)
Not Recovered/not resolved	1 (2.6%)	1 (5.9%)	9 (10.0%)	0 (0%)	13 (15.1%)	24 (10.2%)
Recovered/resolved	11 (28.9%)	2 (11.8%)	26 (28.9%)	2 (40.0%)	33 (38.4%)	74 (31.4%)
Recovering/resolving	3 (7.9%)	0 (0%)	8 (8.9%)	0 (0%)	6 (7.0%)	17 (7.2%)
Unknown	21 (55.3%)	13 (76.5%)	39 (43.3%)	3 (60.0%)	30 (34.9%)	106 (44.9%)
Recovered/resolved with sequelae	0 (0%)	0 (0%)	0 (0%)	0 (0%)	1 (1.2%)	1 (0.4%)

## Data Availability

EV data are publicly available at https://www.adrreports.eu/, accessed on 13 July 2023.

## Supplementary Materials

The following supporting information can be downloaded at: https://www.mdpi.com/article/10.3390/ph17020147/s1. Supplementary Table S1. Other suspected drugs reported in ICSRs of suicidal events with dulaglutide, exenatide, liraglutide, liraglutide/insulin degludec, or semaglutide classified by the Anatomical Therapeutic Chemical (ATC) Classification. Supplementary Table S2. Active ingredients of other suspected drugs reported in ICSRs of suicidal events with dulaglutide, exenatide, liraglutide, liraglutide/insulin degludec, or semaglutide. Supplementary Table S3. Concomitant drugs reported in ICSRs of suicidal events with dulaglutide, exenatide, liraglutide, liraglutide/insulin degludec, or semaglutide classified by the Anatomical Therapeutic Chemical (ATC) Classification. Supplementary Table S4. Active ingredients of concomitant drugs reported in ICSRs of suicidal events with dulaglutide, exenatide, liraglutide, liraglutide/insulin degludec, or semaglutide.
