# Supplementary material for: Glucagon-like Peptide-1 Receptor Agonists and Suicidal Ideation: Analysis of Real-Word Data Collected in the European Pharmacovigilance Database
Source: Pharmaceuticals (Basel). 2024 Jan 23;17(2):147. doi: 10.3390/ph17020147 (PMC10892952; doi:10.3390/ph17020147)
Supplement: Supplementary file 1 [file pharmaceuticals-17-00147-s001.zip › pharmaceuticals-2795683-supplementary.pdf]

Supplementary Table S1. Other suspected drugs reported in ICSRs of suicidal events with dulaglutide, exenatide, liraglutide, liraglutide/insulin degludec, or semaglutide classified by the Anatomical Therapeutic Chemical (ATC) Classification.

ATC	DULAGLUTIDE (N=20)	EXENATIDE (N=41)	LIRAGLUTIDE (N=52)	LIRAGLUTIDE/ INSULIN DEGLUDEC (N=3)	SEMAGLUTIDE (N=8)	OVERALL (N=124)
A10A	13 (65.0%)	0 (0%)	11 (21.2%)	2 (66.7%)	1 (12.5%)	27 (21.8%)
N06A	3 (15.0%)	6 (14.6%)	4 (7.7%)	0 (0%)	1 (12.5%)	14 (11.3%)
A10B	0 (0%)	5 (12.2%)	4 (7.7%)	1 (33.3%)	2 (25.0%)	12 (9.7%)
N03A	0 (0%)	8 (19.5%)	3 (5.8%)	0 (0%)	0 (0%)	11 (8.9%)
C09A	0 (0%)	6 (14.6%)	2 (3.8%)	0 (0%)	0 (0%)	8 (6.5%)
J01X	0 (0%)	8 (19.5%)	0 (0%)	0 (0%)	0 (0%)	8 (6.5%)
N05A	1 (5.0%)	1 (2.4%)	3 (5.8%)	0 (0%)	2 (25.0%)	7 (5.6%)
C10A	0 (0%)	0 (0%)	4 (7.7%)	0 (0%)	0 (0%)	4 (3.2%)
N05C	0 (0%)	0 (0%)	4 (7.7%)	0 (0%)	0 (0%)	4 (3.2%)
C07A	0 (0%)	3 (7.3%)	0 (0%)	0 (0%)	0 (0%)	3 (2.4%)
L01B	0 (0%)	3 (7.3%)	0 (0%)	0 (0%)	0 (0%)	3 (2.4%)
N02B	0 (0%)	0 (0%)	3 (5.8%)	0 (0%)	0 (0%)	3 (2.4%)
N05B	0 (0%)	0 (0%)	2 (3.8%)	0 (0%)	1 (12.5%)	3 (2.4%)
C03C	1 (5.0%)	0 (0%)	1 (1.9%)	0 (0%)	0 (0%)	2 (1.6%)
R06A	0 (0%)	1 (2.4%)	0 (0%)	0 (0%)	1 (12.5%)	2 (1.6%)
D08A	0 (0%)	0 (0%)	2 (3.8%)	0 (0%)	0 (0%)	2 (1.6%)
M03B	0 (0%)	0 (0%)	2 (3.8%)	0 (0%)	0 (0%)	2 (1.6%)
N02A	0 (0%)	0 (0%)	2 (3.8%)	0 (0%)	0 (0%)	2 (1.6%)
N04A	0 (0%)	0 (0%)	2 (3.8%)	0 (0%)	0 (0%)	2 (1.6%)
A07E	1 (5.0%)	0 (0%)	0 (0%)	0 (0%)	0 (0%)	1 (0.8%)
R01B	1 (5.0%)	0 (0%)	0 (0%)	0 (0%)	0 (0%)	1 (0.8%)
A03A	0 (0%)	0 (0%)	1 (1.9%)	0 (0%)	0 (0%)	1 (0.8%)
C09B	0 (0%)	0 (0%)	1 (1.9%)	0 (0%)	0 (0%)	1 (0.8%)
C10B	0 (0%)	0 (0%)	1 (1.9%)	0 (0%)	0 (0%)	1 (0.8%)

A03A: DRUGS FOR FUNCTIONAL GASTROINTESTINAL DISORDERS; A07E: INTESTINAL ANTIINFLAMMATORY AGENTS; A10A: INSULINS AND ANALOGUES; A10B: BLOOD GLUCOSE LOWERING DRUGS, EXCL. INSULINS; C03C: HIGH-CEILING DIURETICS; C07A: BETA BLOCKING AGENTS; C09A: ACE INHIBITORS, PLAIN; C09B: ACE INHIBITORS, COMBINATIONS; C10A: LIPID MODIFYING AGENTS, PLAIN; C10B: LIPID MODIFYING AGENTS, COMBINATIONS; D08A: ANTISEPTICS AND DISINFECTANTS; J01X: OTHER ANTIBACTERIALS; L01B:

ANTIMETABOLITES; M03B: MUSCLE RELAXANTS, CENTRALLY ACTING AGENTS; N02A: OPIOIDS; N02B: OTHER ANALGESICS AND ANTIPYRETICS; N03A: ANTIEPILEPTICS; N04A: ANTICHOLINERGIC AGENTS; N05A: ANTIPSYCHOTICS; N05B: ANXIOLYTICS; N05C: HYPNOTICS AND SEDATIVES; N06A: ANTIDEPRESSANTS; R01B: NASAL DECONGESTANTS FOR SYSTEMIC USE; R06A: ANTIHISTAMINES FOR SYSTEMIC USE.

Supplementary Table S2. Active ingredients of other suspected drugs reported in ICSRs of suicidal events with dulaglutide, exenatide, liraglutide, liraglutide/insulin degludec, or semaglutide.

	DULAGLUTIDE (N=20)	EXENATIDE (N=41)	LIRAGLUTIDE (N=52)	LIRAGLUTIDE/ INSULIN DEGLUDEC (N=3)	SEMAGLUTIDE (N=8)	Overall (N=124)
**SUSPECTS**						
CHLORPHENAMINE MALEATE, PSEUDOEPHEDRINE HYDROCHLORIDE, IBUPROFEN	1 (5.0%)	0 (0%)	0 (0%)	0 (0%)	0 (0%)	1 (0.8%)
DULOXETINE	1 (5.0%)	3 (7.3%)	0 (0%)	0 (0%)	1 (12.5%)	5 (4.0%)
FLUOXETINE	1 (5.0%)	0 (0%)	2 (3.8%)	0 (0%)	0 (0%)	3 (2.4%)
INSULIN ASPART	2 (10.0%)	0 (0%)	3 (5.8%)	2 (66.7%)	0 (0%)	7 (5.6%)
INSULIN DEGLUDEC	3 (15.0%)	0 (0%)	4 (7.7%)	0 (0%)	1 (12.5%)	8 (6.5%)
INSULIN GLARGINE	3 (15.0%)	0 (0%)	4 (7.7%)	0 (0%)	0 (0%)	7 (5.6%)
INSULIN HUMAN	2 (10.0%)	0 (0%)	0 (0%)	0 (0%)	0 (0%)	2 (1.6%)
INSULIN LISPRO	3 (15.0%)	0 (0%)	0 (0%)	0 (0%)	0 (0%)	3 (2.4%)
PREDNISONE	1 (5.0%)	0 (0%)	0 (0%)	0 (0%)	0 (0%)	1 (0.8%)
REPAGLINIDE	1 (5.0%)	0 (0%)	0 (0%)	0 (0%)	0 (0%)	1 (0.8%)
RISPERIDONE	1 (5.0%)	0 (0%)	1 (1.9%)	0 (0%)	0 (0%)	2 (1.6%)
VORTIOXETINE	1 (5.0%)	0 (0%)	0 (0%)	0 (0%)	0 (0%)	1 (0.8%)
ACRIVASTINE, DIPHENHYDRAMINE HYDROCHLORIDE, ZINC ACETATE	0 (0%)	1 (2.4%)	0 (0%)	0 (0%)	0 (0%)	1 (0.8%)
BISOPROLOL	0 (0%)	3 (7.3%)	0 (0%)	0 (0%)	0 (0%)	3 (2.4%)
DAPTOMYCIN	0 (0%)	5 (12.2%)	0 (0%)	0 (0%)	0 (0%)	5 (4.0%)
FLUOROURACIL	0 (0%)	3 (7.3%)	0 (0%)	0 (0%)	0 (0%)	3 (2.4%)
GABAPENTIN	0 (0%)	3 (7.3%)	0 (0%)	0 (0%)	0 (0%)	3 (2.4%)
LISINOPRIL	0 (0%)	6 (14.6%)	2 (3.8%)	0 (0%)	0 (0%)	8 (6.5%)
METFORMIN	0 (0%)	5 (12.2%)	4 (7.7%)	1 (33.3%)	2 (25.0%)	12 (9.7%)
PREGABALIN	0 (0%)	5 (12.2%)	2 (3.8%)	0 (0%)	0 (0%)	7 (5.6%)
QUETIAPINE	0 (0%)	1 (2.4%)	2 (3.8%)	0 (0%)	1 (12.5%)	4 (3.2%)
SERTRALINE	0 (0%)	2 (4.9%)	0 (0%)	0 (0%)	0 (0%)	2 (1.6%)
SERTRALINE HCL	0 (0%)	1 (2.4%)	0 (0%)	0 (0%)	0 (0%)	1 (0.8%)
VANCOMYCIN	0 (0%)	2 (4.9%)	0 (0%)	0 (0%)	0 (0%)	2 (1.6%)

VANCOMYCIN HCL	0 (0%)	1 (2.4%)	0 (0%)	0 (0%)	0 (0%)	1 (0.8%)
ALPRAZOLAM	0 (0%)	0 (0%)	2 (3.8%)	0 (0%)	1 (12.5%)	3 (2.4%)
BIPERIDEN	0 (0%)	0 (0%)	1 (1.9%)	0 (0%)	0 (0%)	1 (0.8%)
BUPROPION	0 (0%)	0 (0%)	2 (3.8%)	0 (0%)	0 (0%)	2 (1.6%)
CYCLOBENZAPRINE	0 (0%)	0 (0%)	2 (3.8%)	0 (0%)	0 (0%)	2 (1.6%)
ESZOPICLONE	0 (0%)	0 (0%)	1 (1.9%)	0 (0%)	0 (0%)	1 (0.8%)
ETHANOL	0 (0%)	0 (0%)	2 (3.8%)	0 (0%)	0 (0%)	2 (1.6%)
FENOFIBRATE, SIMVASTATIN	0 (0%)	0 (0%)	1 (1.9%)	0 (0%)	0 (0%)	1 (0.8%)
FLUNITRAZEPAM	0 (0%)	0 (0%)	1 (1.9%)	0 (0%)	0 (0%)	1 (0.8%)
FUROSEMIDE	0 (0%)	0 (0%)	1 (1.9%)	0 (0%)	0 (0%)	1 (0.8%)
HYDROCODONE BITARTRATE, PARACETAMOL	0 (0%)	0 (0%)	2 (3.8%)	0 (0%)	0 (0%)	2 (1.6%)
MEBEVERINE	0 (0%)	0 (0%)	1 (1.9%)	0 (0%)	0 (0%)	1 (0.8%)
PARACETAMOL	0 (0%)	0 (0%)	3 (5.8%)	0 (0%)	0 (0%)	3 (2.4%)
PERINDOPRIL TERT-BUTYLAMINE, AMLODIPINE	0 (0%)	0 (0%)	1 (1.9%)	0 (0%)	0 (0%)	1 (0.8%)
QUAZEPAM	0 (0%)	0 (0%)	1 (1.9%)	0 (0%)	0 (0%)	1 (0.8%)
ROSUVASTATIN	0 (0%)	0 (0%)	3 (5.8%)	0 (0%)	0 (0%)	3 (2.4%)
ROSUVASTATIN CALCIUM	0 (0%)	0 (0%)	1 (1.9%)	0 (0%)	0 (0%)	1 (0.8%)
TRIAZOLAM	0 (0%)	0 (0%)	1 (1.9%)	0 (0%)	0 (0%)	1 (0.8%)
TRIHEXYPHENIDYL	0 (0%)	0 (0%)	1 (1.9%)	0 (0%)	0 (0%)	1 (0.8%)
VALPROIC ACID	0 (0%)	0 (0%)	1 (1.9%)	0 (0%)	0 (0%)	1 (0.8%)
ARIPIPRAZOLE	0 (0%)	0 (0%)	0 (0%)	0 (0%)	1 (12.5%)	1 (0.8%)
MECLOZINE HYDROCHLORIDE	0 (0%)	0 (0%)	0 (0%)	0 (0%)	1 (12.5%)	1 (0.8%)

Supplementary Table S3. Concomitant drugs reported in ICSRs of suicidal events with dulaglutide, exenatide, liraglutide, liraglutide/insulin degludec, or semaglutide classified by the Anatomical Therapeutic Chemical (ATC) Classification.

ATC	DULAGLUTIDE (N=33)	EXENATIDE (N=116)	LIRAGLUTIDE (N=85)	LIRAGLUTIDE/ INSULIN DEGLUDEC (N=14)	SEMAGLUTIDE (N=115)	OVERALL (N=363)
A10B	13 (39.4%)	7 (6.0%)	10 (11.8%)	2 (14.3%)	17 (14.8%)	49 (13.5%)
N06A	4 (12.1%)	7 (6.0%)	11 (12.9%)	0 (0%)	13 (11.3%)	35 (9.6%)
C10A	1 (3.0%)	8 (6.9%)	6 (7.1%)	2 (14.3%)	8 (7.0%)	25 (6.9%)
A02B	2 (6.1%)	6 (5.2%)	4 (4.7%)	0 (0%)	4 (3.5%)	16 (4.4%)
C09A	1 (3.0%)	3 (2.6%)	5 (5.9%)	2 (14.3%)	4 (3.5%)	15 (4.1%)
N03A	0 (0%)	6 (5.2%)	7 (8.2%)	0 (0%)	2 (1.7%)	15 (4.1%)
N05B	2 (6.1%)	5 (4.3%)	3 (3.5%)	0 (0%)	4 (3.5%)	14 (3.9%)
A10A	3 (9.1%)	4 (3.4%)	0 (0%)	0 (0%)	3 (2.6%)	10 (2.8%)
B01A	0 (0%)	6 (5.2%)	2 (2.4%)	0 (0%)	1 (0.9%)	9 (2.5%)
N02B	0 (0%)	4 (3.4%)	0 (0%)	0 (0%)	5 (4.3%)	9 (2.5%)
C07A	1 (3.0%)	3 (2.6%)	2 (2.4%)	0 (0%)	2 (1.7%)	8 (2.2%)
C08C	0 (0%)	1 (0.9%)	4 (4.7%)	2 (14.3%)	1 (0.9%)	8 (2.2%)
N05A	1 (3.0%)	1 (0.9%)	4 (4.7%)	0 (0%)	1 (0.9%)	7 (1.9%)
A06A	0 (0%)	3 (2.6%)	1 (1.2%)	0 (0%)	2 (1.7%)	6 (1.7%)
A11C	0 (0%)	1 (0.9%)	2 (2.4%)	0 (0%)	3 (2.6%)	6 (1.7%)
B03A	0 (0%)	3 (2.6%)	1 (1.2%)	0 (0%)	2 (1.7%)	6 (1.7%)
R03A	0 (0%)	1 (0.9%)	0 (0%)	0 (0%)	5 (4.3%)	6 (1.7%)
C03C	0 (0%)	5 (4.3%)	0 (0%)	0 (0%)	0 (0%)	5 (1.4%)
C09C	0 (0%)	2 (1.7%)	2 (2.4%)	0 (0%)	1 (0.9%)	5 (1.4%)
G04C	1 (3.0%)	0 (0%)	0 (0%)	0 (0%)	3 (2.6%)	4 (1.1%)
N05C	1 (3.0%)	0 (0%)	2 (2.4%)	0 (0%)	1 (0.9%)	4 (1.1%)
A12B	0 (0%)	4 (3.4%)	0 (0%)	0 (0%)	0 (0%)	4 (1.1%)

H03A	0 (0%)	1 (0.9%)	2 (2.4%)	0 (0%)	1 (0.9%)	4 (1.1%)
R05D	0 (0%)	4 (3.4%)	0 (0%)	0 (0%)	0 (0%)	4 (1.1%)
A11A	0 (0%)	0 (0%)	2 (2.4%)	0 (0%)	2 (1.7%)	4 (1.1%)
B03B	0 (0%)	2 (1.7%)	0 (0%)	0 (0%)	1 (0.9%)	3 (0.8%)
N02A	0 (0%)	2 (1.7%)	1 (1.2%)	0 (0%)	0 (0%)	3 (0.8%)
R06A	0 (0%)	2 (1.7%)	0 (0%)	0 (0%)	1 (0.9%)	3 (0.8%)
V03A	0 (0%)	3 (2.6%)	0 (0%)	0 (0%)	0 (0%)	3 (0.8%)
D03A	0 (0%)	0 (0%)	2 (2.4%)	0 (0%)	1 (0.9%)	3 (0.8%)
R01A	0 (0%)	0 (0%)	1 (1.2%)	0 (0%)	2 (1.7%)	3 (0.8%)
V06D	0 (0%)	0 (0%)	1 (1.2%)	0 (0%)	2 (1.7%)	3 (0.8%)
D11A	0 (0%)	0 (0%)	0 (0%)	0 (0%)	3 (2.6%)	3 (0.8%)
G04B	0 (0%)	0 (0%)	0 (0%)	0 (0%)	3 (2.6%)	3 (0.8%)
G02C	1 (3.0%)	0 (0%)	0 (0%)	0 (0%)	1 (0.9%)	2 (0.6%)
L02A	1 (3.0%)	1 (0.9%)	0 (0%)	0 (0%)	0 (0%)	2 (0.6%)
N04B	1 (3.0%)	0 (0%)	0 (0%)	0 (0%)	1 (0.9%)	2 (0.6%)
B05X	0 (0%)	1 (0.9%)	1 (1.2%)	0 (0%)	0 (0%)	2 (0.6%)
C08D	0 (0%)	1 (0.9%)	0 (0%)	0 (0%)	1 (0.9%)	2 (0.6%)
N06B	0 (0%)	1 (0.9%)	1 (1.2%)	0 (0%)	0 (0%)	2 (0.6%)
S01E	0 (0%)	1 (0.9%)	1 (1.2%)	0 (0%)	0 (0%)	2 (0.6%)
A08A	0 (0%)	0 (0%)	1 (1.2%)	0 (0%)	1 (0.9%)	2 (0.6%)
G03X	0 (0%)	0 (0%)	1 (1.2%)	0 (0%)	1 (0.9%)	2 (0.6%)
C02C	0 (0%)	0 (0%)	0 (0%)	2 (14.3%)	0 (0%)	2 (0.6%)
C03E	0 (0%)	0 (0%)	0 (0%)	2 (14.3%)	0 (0%)	2 (0.6%)
M04A	0 (0%)	0 (0%)	0 (0%)	2 (14.3%)	0 (0%)	2 (0.6%)
A01A	0 (0%)	1 (0.9%)	0 (0%)	0 (0%)	0 (0%)	1 (0.3%)
A04A	0 (0%)	1 (0.9%)	0 (0%)	0 (0%)	0 (0%)	1 (0.3%)
A07E	0 (0%)	1 (0.9%)	0 (0%)	0 (0%)	0 (0%)	1 (0.3%)
A09A	0 (0%)	1 (0.9%)	0 (0%)	0 (0%)	0 (0%)	1 (0.3%)

B05A	0 (0%)	1 (0.9%)	0 (0%)	0 (0%)	0 (0%)	1 (0.3%)
C02D	0 (0%)	1 (0.9%)	0 (0%)	0 (0%)	0 (0%)	1 (0.3%)
C09X	0 (0%)	1 (0.9%)	0 (0%)	0 (0%)	0 (0%)	1 (0.3%)
J01C	0 (0%)	1 (0.9%)	0 (0%)	0 (0%)	0 (0%)	1 (0.3%)
J01D	0 (0%)	1 (0.9%)	0 (0%)	0 (0%)	0 (0%)	1 (0.3%)
J01M	0 (0%)	1 (0.9%)	0 (0%)	0 (0%)	0 (0%)	1 (0.3%)
J05A	0 (0%)	1 (0.9%)	0 (0%)	0 (0%)	0 (0%)	1 (0.3%)
L02B	0 (0%)	1 (0.9%)	0 (0%)	0 (0%)	0 (0%)	1 (0.3%)
L04A	0 (0%)	1 (0.9%)	0 (0%)	0 (0%)	0 (0%)	1 (0.3%)
M01A	0 (0%)	1 (0.9%)	0 (0%)	0 (0%)	0 (0%)	1 (0.3%)
N02C	0 (0%)	1 (0.9%)	0 (0%)	0 (0%)	0 (0%)	1 (0.3%)
R03B	0 (0%)	1 (0.9%)	0 (0%)	0 (0%)	0 (0%)	1 (0.3%)
S01A	0 (0%)	1 (0.9%)	0 (0%)	0 (0%)	0 (0%)	1 (0.3%)
C10B	0 (0%)	0 (0%)	1 (1.2%)	0 (0%)	0 (0%)	1 (0.3%)
G03C	0 (0%)	0 (0%)	1 (1.2%)	0 (0%)	0 (0%)	1 (0.3%)
M03B	0 (0%)	0 (0%)	1 (1.2%)	0 (0%)	0 (0%)	1 (0.3%)
N07B	0 (0%)	0 (0%)	1 (1.2%)	0 (0%)	0 (0%)	1 (0.3%)
S02B	0 (0%)	0 (0%)	1 (1.2%)	0 (0%)	0 (0%)	1 (0.3%)
A03A	0 (0%)	0 (0%)	0 (0%)	0 (0%)	1 (0.9%)	1 (0.3%)
A12C	0 (0%)	0 (0%)	0 (0%)	0 (0%)	1 (0.9%)	1 (0.3%)
A16A	0 (0%)	0 (0%)	0 (0%)	0 (0%)	1 (0.9%)	1 (0.3%)
C01C	0 (0%)	0 (0%)	0 (0%)	0 (0%)	1 (0.9%)	1 (0.3%)
C01D	0 (0%)	0 (0%)	0 (0%)	0 (0%)	1 (0.9%)	1 (0.3%)
C01E	0 (0%)	0 (0%)	0 (0%)	0 (0%)	1 (0.9%)	1 (0.3%)
C09D	0 (0%)	0 (0%)	0 (0%)	0 (0%)	1 (0.9%)	1 (0.3%)
G03A	0 (0%)	0 (0%)	0 (0%)	0 (0%)	1 (0.9%)	1 (0.3%)
G03B	0 (0%)	0 (0%)	0 (0%)	0 (0%)	1 (0.9%)	1 (0.3%)
G03D	0 (0%)	0 (0%)	0 (0%)	0 (0%)	1 (0.9%)	1 (0.3%)

J02A	0 (0%)	0 (0%)	0 (0%)	0 (0%)	1 (0.9%)	1 (0.3%)
R01B	0 (0%)	0 (0%)	0 (0%)	0 (0%)	1 (0.9%)	1 (0.3%)

A01A: STOMATOLOGICAL PREPARATIONS; A02B: DRUGS FOR PEPTIC ULCER AND GASTRO-OESOPHAGEAL REFLUX DISEASE (GORD); A03A: DRUGS FOR FUNCTIONAL GASTROINTESTINAL DISORDERS; A04A: ANTIEMETICS AND ANTINAUSEANTS; A06A: DRUGS FOR CONSTIPATION; A07E: INTESTINAL ANTIINFLAMMATORY AGENTS; A08A: ANTIOBESITY PREPARATIONS, EXCL. DIET PRODUCTS; A09A: DIGESTIVES, INCL. ENZYMES; A10A: INSULINS AND ANALOGUES; A10B: BLOOD GLUCOSE LOWERING DRUGS, EXCL. INSULINS; A11A: MULTIVITAMINS, COMBINATIONS; A11C: VITAMIN A AND D, INCL. COMBINATIONS OF THE TWO; A12B: POTASSIUM; A12C: OTHER MINERAL SUPPLEMENTS; A16A: OTHER ALIMENTARY TRACT AND METABOLISM PRODUCTS; B01A: ANTITHROMBOTIC AGENTS; B03A: IRON PREPARATIONS; B03B: VITAMIN B12 AND FOLIC ACID; B05A: BLOOD AND RELATED PRODUCTS; B05X: I.V. SOLUTION ADDITIVES; C01C: CARDIAC STIMULANTS EXCL. CARDIAC GLYCOSIDES; C01D: VASODILATORS USED IN CARDIAC DISEASES; C01E: OTHER CARDIAC PREPARATIONS; C02C: ANTIADRENERGIC AGENTS, PERIPHERALLY ACTING; C02D: ARTERIOLAR SMOOTH MUSCLE, AGENTS ACTING ON; C03C: HIGH-CEILING DIURETICS; C03E: DIURETICS AND POTASSIUM-SPARING AGENTS IN COMBINATION; C07A: BETA BLOCKING AGENTS; C08C: SELECTIVE CALCIUM CHANNEL BLOCKERS WITH MAINLY VASCULAR EFFECTS; C08D: SELECTIVE CALCIUM CHANNEL BLOCKERS WITH DIRECT CARDIAC EFFECTS; C09A: ACE INHIBITORS, PLAIN; C09C: ANGIOTENSIN II RECEPTOR BLOCKERS (ARBs), PLAIN; C09D: ANGIOTENSIN II RECEPTOR BLOCKERS (ARBs), COMBINATIONS; C09X: OTHER AGENTS ACTING ON THE RENIN-ANGIOTENSIN SYSTEM; C10A: LIPID MODIFYING AGENTS, PLAIN; C10B: LIPID MODIFYING AGENTS, COMBINATIONS; D03A: CICATRIZANTS; D11A: OTHER DERMATOLOGICAL PREPARATIONS; G02C: OTHER GYNECOLOGICALS; G03A: HORMONAL CONTRACEPTIVES FOR SYSTEMIC USE; G03B: ANDROGENS; G03C: ESTROGENS; G03D: PROGESTOGENS; G03X: OTHER SEX HORMONES AND MODULATORS OF THE GENITAL SYSTEM; G04B: UROLOGICALS; G04C: DRUGS USED IN BENIGN PROSTATIC HYPERTROPHY; H03A: THYROID PREPARATIONS; J01C: BETA-LACTAM ANTIBACTERIALS, PENICILLINS; J01D: OTHER BETA-LACTAM ANTIBACTERIALS; J01M: QUINOLONE ANTIBACTERIALS; J02A: ANTIMYCOTICS FOR SYSTEMIC USE; J05A: DIRECT ACTING ANTIVIRALS; L02A: HORMONES AND RELATED AGENTS; L02B: HORMONE ANTAGONISTS AND RELATED AGENTS; L04A: IMMUNOSUPPRESSANTS; M01A: ANTIINFLAMMATORY AND ANTIRHEUMATIC PRODUCTS, NON-STEROIDS; M03B: MUSCLE RELAXANTS, CENTRALLY ACTING AGENTS; M04A: ANTIGOUT PREPARATIONS; N02A: OPIOIDS; N02B: OTHER ANALGESICS AND ANTIPYRETICS; N02C: ANTIMIGRAINE PREPARATIONS; N03A: ANTIEPILEPTICS; N04B: DOPAMINERGIC AGENTS; N05A: ANTIPSYCHOTICS; N05B: ANXIOLYTICS; N05C: HYPNOTICS AND SEDATIVES; N06A: ANTIDEPRESSANTS; N06B: PSYCHOSTIMULANTS, AGENTS USED FOR ADHD AND NOOTROPICS; N07B: DRUGS USED IN ADDICTIVE DISORDERS; R01A: DECONGESTANTS AND OTHER NASAL PREPARATIONS FOR TOPICAL USE; R01B: NASAL DECONGESTANTS FOR SYSTEMIC USE; R03A: ADRENERGICS, INHALANTS; R03B: OTHER DRUGS FOR OBSTRUCTIVE AIRWAY DISEASES, INHALANTS; R05D: COUGH SUPPRESSANTS, EXCL. COMBINATIONS WITH EXPECTORANTS; R06A: ANTIHISTAMINES FOR SYSTEMIC USE; S01A: ANTIINFECTIVES; S01E: ANTIGLAUCOMA PREPARATIONS AND MIOTICS; S02B: CORTICOSTEROIDS; V03A: ALL OTHER THERAPEUTIC PRODUCTS; V06D: OTHER NUTRIENTS.

Supplementary Table S4. Active ingredients of concomitant drugs reported in ICSRs of suicidal events with dulaglutide, exenatide, liraglutide, liraglutide/insulin degludec, or semaglutide.

	DULAGLUTIDE (N=33)	EXENATIDE (N=116)	LIRAGLUTIDE (N=85)	LIRAGLUTIDE/ INSULIN DEGLUDEC (N=14)	SEMAGLUTIDE (N=115)	OVERAL L (N=363)
**CONCOMITANTS**						
ALPRAZOLAM	1 (3.0%)	1 (0.9%)	0 (0%)	0 (0%)	1 (0.9%)	3 (0.8%)
BISOPROLOL	1 (3.0%)	0 (0%)	0 (0%)	0 (0%)	2 (1.7%)	3 (0.8%)
BROMAZEPAM	1 (3.0%)	0 (0%)	0 (0%)	0 (0%)	0 (0%)	1 (0.3%)
CAPTOPRIL	1 (3.0%)	0 (0%)	0 (0%)	0 (0%)	0 (0%)	1 (0.3%)
CITALOPRAM	1 (3.0%)	0 (0%)	0 (0%)	0 (0%)	1 (0.9%)	2 (0.6%)
DAPAGLIFLOZIN	2 (6.1%)	0 (0%)	0 (0%)	0 (0%)	0 (0%)	2 (0.6%)
DULOXETINE	1 (3.0%)	1 (0.9%)	2 (2.4%)	0 (0%)	0 (0%)	4 (1.1%)
EMPAGLIFLOZIN	1 (3.0%)	0 (0%)	0 (0%)	0 (0%)	1 (0.9%)	2 (0.6%)
FENOFIBRATE	1 (3.0%)	1 (0.9%)	0 (0%)	0 (0%)	0 (0%)	2 (0.6%)
FLUNITRAZEPAM	1 (3.0%)	0 (0%)	1 (1.2%)	0 (0%)	0 (0%)	2 (0.6%)
GLIBENCLAMIDE	1 (3.0%)	0 (0%)	0 (0%)	0 (0%)	1 (0.9%)	2 (0.6%)
INSULIN GLARGINE	2 (6.1%)	2 (1.7%)	0 (0%)	0 (0%)	1 (0.9%)	5 (1.4%)
INSULIN LISPRO	1 (3.0%)	1 (0.9%)	0 (0%)	0 (0%)	0 (0%)	2 (0.6%)
LANSOPRAZOLE	1 (3.0%)	0 (0%)	2 (2.4%)	0 (0%)	2 (1.7%)	5 (1.4%)
LEUPRORELIN	1 (3.0%)	1 (0.9%)	0 (0%)	0 (0%)	0 (0%)	2 (0.6%)
LIRAGLUTIDE	1 (3.0%)	0 (0%)	0 (0%)	0 (0%)	0 (0%)	1 (0.3%)
LUSEOGLIFLOZIN	1 (3.0%)	0 (0%)	0 (0%)	0 (0%)	0 (0%)	1 (0.3%)
MAPROTILINE	1 (3.0%)	0 (0%)	0 (0%)	0 (0%)	0 (0%)	1 (0.3%)
METFORMIN	5 (15.2%)	2 (1.7%)	6 (7.1%)	1 (7.1%)	9 (7.8%)	23 (6.3%)
NAPROXEN	1 (3.0%)	0 (0%)	0 (0%)	0 (0%)	1 (0.9%)	2 (0.6%)
OMEPRAZOLE	1 (3.0%)	1 (0.9%)	1 (1.2%)	0 (0%)	2 (1.7%)	5 (1.4%)
PIOGLITAZONE	1 (3.0%)	1 (0.9%)	0 (0%)	0 (0%)	0 (0%)	2 (0.6%)
PRAMIPEXOLE	1 (3.0%)	0 (0%)	0 (0%)	0 (0%)	1 (0.9%)	2 (0.6%)
QUETIAPINE	1 (3.0%)	0 (0%)	1 (1.2%)	0 (0%)	0 (0%)	2 (0.6%)
SERTRALINE	1 (3.0%)	1 (0.9%)	0 (0%)	0 (0%)	3 (2.6%)	5 (1.4%)
SITAGLIPTIN	1 (3.0%)	0 (0%)	0 (0%)	0 (0%)	0 (0%)	1 (0.3%)
TAMSULOSIN	1 (3.0%)	0 (0%)	0 (0%)	0 (0%)	2 (1.7%)	3 (0.8%)
ACETYLSALICYLIC ACID	0 (0%)	3 (2.6%)	0 (0%)	0 (0%)	3 (2.6%)	6 (1.7%)
ALBUMIN	0 (0%)	1 (0.9%)	0 (0%)	0 (0%)	0 (0%)	1 (0.3%)
ALISKIREN	0 (0%)	1 (0.9%)	0 (0%)	0 (0%)	0 (0%)	1 (0.3%)
ALOGLIPTIN	0 (0%)	1 (0.9%)	0 (0%)	0 (0%)	0 (0%)	1 (0.3%)
AMLODIPINE	0 (0%)	1 (0.9%)	4 (4.7%)	0 (0%)	1 (0.9%)	6 (1.7%)

ARIPIPRAZOLE	0 (0%)	1 (0.9%)	1 (1.2%)	0 (0%)	0 (0%)	2 (0.6%)
ATORVASTATIN	0 (0%)	2 (1.7%)	3 (3.5%)	2 (14.3%)	4 (3.5%)	11 (3.0%)
AZITHROMYCIN	0 (0%)	1 (0.9%)	0 (0%)	0 (0%)	0 (0%)	1 (0.3%)
BENAZEPRIL	0 (0%)	1 (0.9%)	0 (0%)	0 (0%)	0 (0%)	1 (0.3%)
BICALUTAMIDE	0 (0%)	1 (0.9%)	0 (0%)	0 (0%)	0 (0%)	1 (0.3%)
BUPROPION	0 (0%)	1 (0.9%)	2 (2.4%)	0 (0%)	3 (2.6%)	6 (1.7%)
CANNABIDIOL	0 (0%)	1 (0.9%)	0 (0%)	0 (0%)	0 (0%)	1 (0.3%)
CARVEDILOL	0 (0%)	1 (0.9%)	0 (0%)	0 (0%)	0 (0%)	1 (0.3%)
CEFAZOLIN	0 (0%)	1 (0.9%)	0 (0%)	0 (0%)	0 (0%)	1 (0.3%)
CETIRIZINE	0 (0%)	2 (1.7%)	0 (0%)	0 (0%)	1 (0.9%)	3 (0.8%)
CIPROFLOXACIN	0 (0%)	1 (0.9%)	0 (0%)	0 (0%)	0 (0%)	1 (0.3%)
CLONAZEPAM	0 (0%)	1 (0.9%)	1 (1.2%)	0 (0%)	0 (0%)	2 (0.6%)
COLECALCIFEROL	0 (0%)	1 (0.9%)	1 (1.2%)	0 (0%)	1 (0.9%)	3 (0.8%)
CYANOCOBALAMIN	0 (0%)	1 (0.9%)	0 (0%)	0 (0%)	1 (0.9%)	2 (0.6%)
DEXAMFETAMINE	0 (0%)	1 (0.9%)	0 (0%)	0 (0%)	0 (0%)	1 (0.3%)
DILTIAZEM	0 (0%)	1 (0.9%)	0 (0%)	0 (0%)	1 (0.9%)	2 (0.6%)
DOCUSATE	0 (0%)	1 (0.9%)	0 (0%)	0 (0%)	0 (0%)	1 (0.3%)
ESCITALOPRAM	0 (0%)	2 (1.7%)	2 (2.4%)	0 (0%)	2 (1.7%)	6 (1.7%)
EZETIMIBE	0 (0%)	2 (1.7%)	0 (0%)	0 (0%)	0 (0%)	2 (0.6%)
FERROUS SULFATE	0 (0%)	2 (1.7%)	1 (1.2%)	0 (0%)	0 (0%)	3 (0.8%)
FISH OIL	0 (0%)	1 (0.9%)	0 (0%)	0 (0%)	2 (1.7%)	3 (0.8%)
FLUOXETINE	0 (0%)	2 (1.7%)	2 (2.4%)	0 (0%)	0 (0%)	4 (1.1%)
FLUTICASONE, SALMETEROL	0 (0%)	1 (0.9%)	0 (0%)	0 (0%)	1 (0.9%)	2 (0.6%)
FUROSEMIDE	0 (0%)	1 (0.9%)	0 (0%)	0 (0%)	0 (0%)	1 (0.3%)
GABAPENTIN	0 (0%)	4 (3.4%)	2 (2.4%)	0 (0%)	0 (0%)	6 (1.7%)
GLIMEPIRIDE	0 (0%)	2 (1.7%)	0 (0%)	0 (0%)	0 (0%)	2 (0.6%)
GLIPIZIDE	0 (0%)	1 (0.9%)	0 (0%)	0 (0%)	1 (0.9%)	2 (0.6%)
GLUCOSE, MAGNESIUM, SODIUM CHLORIDE, CALCIUM	0 (0%)	1 (0.9%)	0 (0%)	0 (0%)	0 (0%)	1 (0.3%)
HEPARIN	0 (0%)	1 (0.9%)	0 (0%)	0 (0%)	0 (0%)	1 (0.3%)
HYDRALAZINE	0 (0%)	1 (0.9%)	0 (0%)	0 (0%)	0 (0%)	1 (0.3%)
HYDROCODONE	0 (0%)	4 (3.4%)	0 (0%)	0 (0%)	0 (0%)	4 (1.1%)
HYDROXYZINE	0 (0%)	2 (1.7%)	0 (0%)	0 (0%)	0 (0%)	2 (0.6%)
INFLIXIMAB	0 (0%)	1 (0.9%)	0 (0%)	0 (0%)	0 (0%)	1 (0.3%)
INSULIN	0 (0%)	1 (0.9%)	0 (0%)	0 (0%)	0 (0%)	1 (0.3%)
IPRATROPIUM	0 (0%)	1 (0.9%)	0 (0%)	0 (0%)	0 (0%)	1 (0.3%)
IRBESARTAN	0 (0%)	1 (0.9%)	0 (0%)	0 (0%)	0 (0%)	1 (0.3%)
IRON	0 (0%)	1 (0.9%)	0 (0%)	0 (0%)	1 (0.9%)	2 (0.6%)
LABETALOL	0 (0%)	1 (0.9%)	0 (0%)	0 (0%)	0 (0%)	1 (0.3%)

LACTULOSE	0 (0%)	1 (0.9%)	0 (0%)	0 (0%)	0 (0%)	1 (0.3%)
LEVOTHYROXINE	0 (0%)	1 (0.9%)	1 (1.2%)	0 (0%)	0 (0%)	2 (0.6%)
LISINOPRIL	0 (0%)	2 (1.7%)	0 (0%)	0 (0%)	1 (0.9%)	3 (0.8%)
LORAZEPAM	0 (0%)	2 (1.7%)	1 (1.2%)	0 (0%)	1 (0.9%)	4 (1.1%)
LOSARTAN	0 (0%)	1 (0.9%)	0 (0%)	0 (0%)	1 (0.9%)	2 (0.6%)
MACROGOL 3350	0 (0%)	1 (0.9%)	0 (0%)	0 (0%)	1 (0.9%)	2 (0.6%)
MELOXICAM	0 (0%)	1 (0.9%)	0 (0%)	0 (0%)	0 (0%)	1 (0.3%)
METOPROLOL	0 (0%)	1 (0.9%)	1 (1.2%)	0 (0%)	0 (0%)	2 (0.6%)
METRONIDAZOLE	0 (0%)	1 (0.9%)	0 (0%)	0 (0%)	0 (0%)	1 (0.3%)
MORPHINE	0 (0%)	1 (0.9%)	0 (0%)	0 (0%)	0 (0%)	1 (0.3%)
NALOXONE	0 (0%)	2 (1.7%)	0 (0%)	0 (0%)	0 (0%)	2 (0.6%)
OMEPRAZOLE, PANTOPRAZOLE	0 (0%)	2 (1.7%)	0 (0%)	0 (0%)	0 (0%)	2 (0.6%)
ONDANSETRON	0 (0%)	1 (0.9%)	0 (0%)	0 (0%)	0 (0%)	1 (0.3%)
OXYCODONE, PARACETAMOL	0 (0%)	1 (0.9%)	0 (0%)	0 (0%)	0 (0%)	1 (0.3%)
PANCREALIPASE	0 (0%)	1 (0.9%)	0 (0%)	0 (0%)	0 (0%)	1 (0.3%)
PANTOPRAZOLE	0 (0%)	1 (0.9%)	0 (0%)	0 (0%)	0 (0%)	1 (0.3%)
PARACETAMOL	0 (0%)	1 (0.9%)	0 (0%)	0 (0%)	2 (1.7%)	3 (0.8%)
PIPERACILLIN, TAZOBACTAM	0 (0%)	1 (0.9%)	0 (0%)	0 (0%)	0 (0%)	1 (0.3%)
POTASSIUM	0 (0%)	4 (3.4%)	0 (0%)	0 (0%)	0 (0%)	4 (1.1%)
PRAVASTATIN	0 (0%)	1 (0.9%)	1 (1.2%)	0 (0%)	0 (0%)	2 (0.6%)
PREDNISONE	0 (0%)	1 (0.9%)	0 (0%)	0 (0%)	0 (0%)	1 (0.3%)
RABEPRAZOLE	0 (0%)	1 (0.9%)	1 (1.2%)	0 (0%)	0 (0%)	2 (0.6%)
RANITIDINE	0 (0%)	1 (0.9%)	0 (0%)	0 (0%)	0 (0%)	1 (0.3%)
RIVAROXABAN	0 (0%)	1 (0.9%)	0 (0%)	0 (0%)	0 (0%)	1 (0.3%)
SEVELAMER	0 (0%)	1 (0.9%)	0 (0%)	0 (0%)	0 (0%)	1 (0.3%)
SIMVASTATIN	0 (0%)	1 (0.9%)	1 (1.2%)	0 (0%)	1 (0.9%)	3 (0.8%)
SUMATRIPTAN	0 (0%)	1 (0.9%)	0 (0%)	0 (0%)	0 (0%)	1 (0.3%)
TIMOLOL, BRIMONIDINE	0 (0%)	1 (0.9%)	0 (0%)	0 (0%)	0 (0%)	1 (0.3%)
TORASEMIDE	0 (0%)	4 (3.4%)	0 (0%)	0 (0%)	0 (0%)	4 (1.1%)
VALACICLOVIR	0 (0%)	1 (0.9%)	0 (0%)	0 (0%)	0 (0%)	1 (0.3%)
VITAMIN B12	0 (0%)	1 (0.9%)	0 (0%)	0 (0%)	0 (0%)	1 (0.3%)
WARFARIN	0 (0%)	4 (3.4%)	1 (1.2%)	0 (0%)	0 (0%)	5 (1.4%)
ARGININE ASPARTATE	0 (0%)	0 (0%)	1 (1.2%)	0 (0%)	0 (0%)	1 (0.3%)
ATENOLOL	0 (0%)	0 (0%)	1 (1.2%)	0 (0%)	0 (0%)	1 (0.3%)
integratore	0 (0%)	0 (0%)	1 (1.2%)	0 (0%)	0 (0%)	1 (0.3%)
CLOPIDOGREL	0 (0%)	0 (0%)	1 (1.2%)	0 (0%)	0 (0%)	1 (0.3%)
COD-LIVER OIL	0 (0%)	0 (0%)	2 (2.4%)	0 (0%)	1 (0.9%)	3 (0.8%)
CORTICOSTEROID NOS	0 (0%)	0 (0%)	1 (1.2%)	0 (0%)	0 (0%)	1 (0.3%)

CYCLOBENZAPRINE	0 (0%)	0 (0%)	1 (1.2%)	0 (0%)	0 (0%)	1 (0.3%)
DIAZEPAM	0 (0%)	0 (0%)	1 (1.2%)	0 (0%)	1 (0.9%)	2 (0.6%)
ENALAPRIL	0 (0%)	0 (0%)	1 (1.2%)	0 (0%)	0 (0%)	1 (0.3%)
ESTRADIOL	0 (0%)	0 (0%)	1 (1.2%)	0 (0%)	0 (0%)	1 (0.3%)
ETIZOLAM	0 (0%)	0 (0%)	1 (1.2%)	0 (0%)	0 (0%)	1 (0.3%)
GLICLAZIDE	0 (0%)	0 (0%)	1 (1.2%)	0 (0%)	4 (3.5%)	5 (1.4%)
HYDROCODONE, PARACETAMOL	0 (0%)	0 (0%)	1 (1.2%)	0 (0%)	0 (0%)	1 (0.3%)
LAMOTRIGINE	0 (0%)	0 (0%)	1 (1.2%)	0 (0%)	0 (0%)	1 (0.3%)
LATANOPROST	0 (0%)	0 (0%)	1 (1.2%)	0 (0%)	0 (0%)	1 (0.3%)
LEVOTHYROXINE SODIUM	0 (0%)	0 (0%)	1 (1.2%)	0 (0%)	1 (0.9%)	2 (0.6%)
MITIGLINIDE	0 (0%)	0 (0%)	1 (1.2%)	0 (0%)	0 (0%)	1 (0.3%)
MODAFINIL	0 (0%)	0 (0%)	1 (1.2%)	0 (0%)	0 (0%)	1 (0.3%)
NICOTINE	0 (0%)	0 (0%)	1 (1.2%)	0 (0%)	0 (0%)	1 (0.3%)
OLANZAPINE	0 (0%)	0 (0%)	1 (1.2%)	0 (0%)	0 (0%)	1 (0.3%)
ORLISTAT	0 (0%)	0 (0%)	1 (1.2%)	0 (0%)	0 (0%)	1 (0.3%)
PAROXETINE	0 (0%)	0 (0%)	3 (3.5%)	0 (0%)	0 (0%)	3 (0.8%)
PERINDOPRIL	0 (0%)	0 (0%)	4 (4.7%)	0 (0%)	2 (1.7%)	6 (1.7%)
PRASTERONE	0 (0%)	0 (0%)	1 (1.2%)	0 (0%)	1 (0.9%)	2 (0.6%)
PREDNISOLONE	0 (0%)	0 (0%)	1 (1.2%)	0 (0%)	0 (0%)	1 (0.3%)
PREGABALIN	0 (0%)	0 (0%)	1 (1.2%)	0 (0%)	2 (1.7%)	3 (0.8%)
REPAGLINIDE	0 (0%)	0 (0%)	1 (1.2%)	0 (0%)	0 (0%)	1 (0.3%)
ROSUVASTATIN	0 (0%)	0 (0%)	1 (1.2%)	0 (0%)	1 (0.9%)	2 (0.6%)
SENNA ALEXANDRIAN	0 (0%)	0 (0%)	1 (1.2%)	0 (0%)	0 (0%)	1 (0.3%)
SIMVASTATIN, EZETIMIBE	0 (0%)	0 (0%)	1 (1.2%)	0 (0%)	0 (0%)	1 (0.3%)
SULPIRIDE	0 (0%)	0 (0%)	1 (1.2%)	0 (0%)	0 (0%)	1 (0.3%)
TELMISARTAN	0 (0%)	0 (0%)	2 (2.4%)	0 (0%)	0 (0%)	2 (0.6%)
TEMAZEPAM	0 (0%)	0 (0%)	1 (1.2%)	0 (0%)	0 (0%)	1 (0.3%)
TOPIRAMATE	0 (0%)	0 (0%)	2 (2.4%)	0 (0%)	0 (0%)	2 (0.6%)
VILDAGLIPTIN	0 (0%)	0 (0%)	1 (1.2%)	0 (0%)	0 (0%)	1 (0.3%)
VITAMIN D	0 (0%)	0 (0%)	1 (1.2%)	0 (0%)	1 (0.9%)	2 (0.6%)
VITAMINS NOS	0 (0%)	0 (0%)	2 (2.4%)	0 (0%)	1 (0.9%)	3 (0.8%)
ALLOPURINOL	0 (0%)	0 (0%)	0 (0%)	2 (14.3%)	0 (0%)	2 (0.6%)
ALTIZIDE, SPIRONOLACTONE	0 (0%)	0 (0%)	0 (0%)	2 (14.3%)	0 (0%)	2 (0.6%)
IPRAGLIFLOZIN	0 (0%)	0 (0%)	0 (0%)	1 (7.1%)	0 (0%)	1 (0.3%)
LERCANIDIPINE	0 (0%)	0 (0%)	0 (0%)	2 (14.3%)	0 (0%)	2 (0.6%)
RAMIPRIL	0 (0%)	0 (0%)	0 (0%)	2 (14.3%)	1 (0.9%)	3 (0.8%)
URAPIDIL	0 (0%)	0 (0%)	0 (0%)	2 (14.3%)	0 (0%)	2 (0.6%)
ALLIUM SATIVUM	0 (0%)	0 (0%)	0 (0%)	0 (0%)	1 (0.9%)	1 (0.3%)

AMISULPRIDE	0 (0%)	0 (0%)	0 (0%)	0 (0%)	1 (0.9%)	1 (0.3%)
ASCORBIC ACID, CALCIUM GLUBIONATE, CALCIUM CARBONATE, COLECALCIFEROL, CALCIUM	0 (0%)	0 (0%)	0 (0%)	0 (0%)	1 (0.9%)	1 (0.3%)
BUDESONIDE, FORMOTEROL	0 (0%)	0 (0%)	0 (0%)	0 (0%)	1 (0.9%)	1 (0.3%)
DEXLANSOPRAZOLE, LANSOPRAZOLE	0 (0%)	0 (0%)	0 (0%)	0 (0%)	1 (0.9%)	1 (0.3%)
DICLOFENAC	0 (0%)	0 (0%)	0 (0%)	0 (0%)	2 (1.7%)	2 (0.6%)
DOCUSATE SODIUM	0 (0%)	0 (0%)	0 (0%)	0 (0%)	1 (0.9%)	1 (0.3%)
DROSPIRENONE, ETHINYLESTRADIOL	0 (0%)	0 (0%)	0 (0%)	0 (0%)	1 (0.9%)	1 (0.3%)
EXENATIDE	0 (0%)	0 (0%)	0 (0%)	0 (0%)	1 (0.9%)	1 (0.3%)
FINASTERIDE	0 (0%)	0 (0%)	0 (0%)	0 (0%)	1 (0.9%)	1 (0.3%)
FLUTICASONE	0 (0%)	0 (0%)	0 (0%)	0 (0%)	1 (0.9%)	1 (0.3%)
HEME IRON POLYPEPTIDE	0 (0%)	0 (0%)	0 (0%)	0 (0%)	1 (0.9%)	1 (0.3%)
HYDROCHLOROTHIAZIDE, LOSARTAN	0 (0%)	0 (0%)	0 (0%)	0 (0%)	1 (0.9%)	1 (0.3%)
INSULIN DEGLUDEC	0 (0%)	0 (0%)	0 (0%)	0 (0%)	2 (1.7%)	2 (0.6%)
ISOSORBIDE	0 (0%)	0 (0%)	0 (0%)	0 (0%)	1 (0.9%)	1 (0.3%)
KETOCONAZOLE	0 (0%)	0 (0%)	0 (0%)	0 (0%)	1 (0.9%)	1 (0.3%)
MAGNESIUM GLYCINATE	0 (0%)	0 (0%)	0 (0%)	0 (0%)	1 (0.9%)	1 (0.3%)
MEBEVERINE	0 (0%)	0 (0%)	0 (0%)	0 (0%)	1 (0.9%)	1 (0.3%)
MEXAZOLAM	0 (0%)	0 (0%)	0 (0%)	0 (0%)	1 (0.9%)	1 (0.3%)
MIRABEGRON	0 (0%)	0 (0%)	0 (0%)	0 (0%)	1 (0.9%)	1 (0.3%)
MIRTAZAPINE	0 (0%)	0 (0%)	0 (0%)	0 (0%)	1 (0.9%)	1 (0.3%)
MOMETASONE	0 (0%)	0 (0%)	0 (0%)	0 (0%)	1 (0.9%)	1 (0.3%)
NORTRIPTYLINE	0 (0%)	0 (0%)	0 (0%)	0 (0%)	1 (0.9%)	1 (0.3%)
PHENTERMINE	0 (0%)	0 (0%)	0 (0%)	0 (0%)	1 (0.9%)	1 (0.3%)
PHENYLEPHRINE	0 (0%)	0 (0%)	0 (0%)	0 (0%)	1 (0.9%)	1 (0.3%)
PIMECROLIMUS	0 (0%)	0 (0%)	0 (0%)	0 (0%)	1 (0.9%)	1 (0.3%)
PROGESTERONE	0 (0%)	0 (0%)	0 (0%)	0 (0%)	1 (0.9%)	1 (0.3%)
PSEUDOEPHEDRINE	0 (0%)	0 (0%)	0 (0%)	0 (0%)	1 (0.9%)	1 (0.3%)
SALBUTAMOL	0 (0%)	0 (0%)	0 (0%)	0 (0%)	2 (1.7%)	2 (0.6%)
SILDENAFIL	0 (0%)	0 (0%)	0 (0%)	0 (0%)	1 (0.9%)	1 (0.3%)
TADALAFIL	0 (0%)	0 (0%)	0 (0%)	0 (0%)	1 (0.9%)	1 (0.3%)
TESTOSTERONE CIPIONATE	0 (0%)	0 (0%)	0 (0%)	0 (0%)	1 (0.9%)	1 (0.3%)
TICAGRELOR	0 (0%)	0 (0%)	0 (0%)	0 (0%)	1 (0.9%)	1 (0.3%)
TURMERIC	0 (0%)	0 (0%)	0 (0%)	0 (0%)	1 (0.9%)	1 (0.3%)

UBIDECARENONE	0 (0%)	0 (0%)	0 (0%)	0 (0%)	1 (0.9%)	1 (0.3%)
UMECLIDINIUM, FLUTICASONE, VILANTEROL	0 (0%)	0 (0%)	0 (0%)	0 (0%)	1 (0.9%)	1 (0.3%)
VENLAFAXINE	0 (0%)	0 (0%)	0 (0%)	0 (0%)	2 (1.7%)	2 (0.6%)
ZINC ACETATE	0 (0%)	0 (0%)	0 (0%)	0 (0%)	1 (0.9%)	1 (0.3%)
ZOLPIDEM	0 (0%)	0 (0%)	0 (0%)	0 (0%)	1 (0.9%)	1 (0.3%)
